# The Professional Fees of Physicians and Surgeons

**Published:** 1916-11-25

**Authors:** 


### The Hospital

The Workers' Newspaper of Administrative Medicine and Institutional Life, Administration, National Insurance and Health.

No. 1590, Vol. LXI. SATURDAY, NOVEMBER 25, 1916. PRICE ONE PENNY.

### THE PROFESSIONAL FEES OF PHYSICIANS AND SURGEONS.

The fixing of the fee to be paid for professional services is almost inevitably a matter of some delicacy and difficulty, as between expert advice on the one hand and hard cash on the other there is no common measure. The adviser himself professes to be moved by other than commercial considerations, and the beneficiary receives something which can bring him no return when tested in the open market. Hence the ordinary means of estimating values are not available. There remains only the arrangement which, as a matter of fact, actually exists. The expert fixes the fee which he regards as reasonable, and it is for the member of the public who wishes direction to decide whether it is or is not worth while to pay this fee. But even experts are not free from the pressure of competition, and under this most of them, at all events, have to subdue personal inclination to the level of custom as this is established by their fellows. The very exceptional authority, or the person believed by the seeking public to be a very exceptional authority, may succeed in defying this custom, but for the majority there is no escape. Even in these lofty and select circles the struggle for existence, or it may be the struggle for luxuries, exerts its sway. Such, then, are the influences under which the fees charged and paid for medical services—as for other professional services—are determined, qualified by the honourable tradition of the profession which demands that, so far as possible, poverty shall not deprive the poor man of efficient aid.

This question of fees, in so far at least as it relates to the amounts paid to physicians and surgeons respectively, has often been a topic of private conversation in professional circles. It has, however, recently been raised in a more public fashion by a letter written by the late Sir Lauder Brunton, and published since his death. The text of the letter is that, while the physician who, as a result of "trained brainwork," judges and decides that an operation is necessary receives only a modest fee of three guineas, the surgeon, who provides "the mere mechanical work," is paid more than thirty times this sum. So far as the terms of the letter are concerned, the writer's conclusion seems to be, not that the physician should be paid more but that the surgeon should be paid less. At least, he does not plainly advance the former proposition, though it is perhaps implicit in his letter. This proposition, at all events, is the claim of many physicians; and no less an authority than Dr. Mercier says plainly that "the fees of physicians, the higher branch of the profession, are inadequate," while he is equally confident that the fees paid to surgeons are excessive. The two proposals have no necessary connection the one with the other, and they might well be argued apart. Nor is the force of the physician's claim likely to be strengthened by attempts to belittle the work of the surgeon. Is it, for example, fair to speak of this work as "merely mechanical," and as opposed to "trained brainwork," or to hold up to scorn a skilled operator on adenoid growths in the naso-pharynx as a "surgeon who scratches the back of a child's throat with his finger-nail" for a fee of thirty guineas? We venture to say that the case for the physicians will not be helped by arguments of this order, and that a scornful public will be inclined to attribute them to an unworthy jealousy. It may be trying to see so much surgical prosperity, but the physician will hardly advance his own position by deriding the causes of this prosperity. Boswell, so it is said, once confessed to Johnson that he sometimes felt a mean-spirited fellow. "So do I," responded the sage, "but I never tell anyone."

On the direct issue relative to the actual fees paid to consulting physicians we quite agree that these are inadequate and ought to be increased. The reason for this conclusion we find, not by comparison with any fancied or arbitrary standard or with the fees paid to surgeons, but by a much less controversial method. The proof of the conclusion is found for us in the experience that these fees mean an income which is failing to attract the younger members of the profession, so that inevitably ere long the consulting physicians will decline in numbers. This, in our opinion, would mean a position disadvantageous for the public interest. On this large ground it is therefore fair to say that the physician ought to be paid more liberally, for otherwise he will not be preserved. Capacity goes in the long run where the best remuneration is offered, and the recent graduate of to-day has constantly before his eyes the broad fact that surgery or some of the more specialised branches of practice have more abundant rewards and more prompt rewards than those offered by medicine. The same story is told in every medical school in the country, and the influence of it is well known to those who follow the career of the best and most promising of our medical students. There are exceptions, of course, but the general rule holds good, and it holds good to an increasing extent and over a larger area. The remedy obviously is to teach the public to value more highly the opinion and advice of the physician. There is only one way to secure this. The hindrance is provided by the senior men. So long as men bearing titular and other distinctions continue to ask a minimum fee so long will the present state of matters continue. In such circumstances the juniors are obviously helpless and the bad tradition repeats itself. Thus while our sympathies are with the physicians' claim, the complaints at least of senior men do not touch us. When they have garnished their own house it will be time enough to sweep clean that of the surgeon.

